# Maternal Vitamin D Status in Preeclampsia: Seasonal Changes Are Not Influenced by Placental Gene Expression of Vitamin D Metabolizing Enzymes

**DOI:** 10.1371/journal.pone.0105558

**Published:** 2014-08-22

**Authors:** Carolin Lechtermann, Berthold P. Hauffa, Ralf Herrmann, Michael M. Schündeln, Alexandra Gellhaus, Markus Schmidt, Corinna Grasemann

**Affiliations:** 1 Department of Pediatric Endocrinology and Diabetology, Kinderklinik II, Universitätsklinikum-Essen and the University of Duisburg-Essen, Essen, Germany; 2 Department of Neonatology, Kinderklinik I, Universitätsklinikum -Essen and the University of Duisburg-Essen, Essen, Germany; 3 Department of Pediatric Oncology and Hematology, Kinderklinik III, Universitätsklinikum -Essen and the University of Duisburg-Essen, Essen, Germany; 4 Institute of Molecular Biology, Universitätsklinikum -Essen and the University of Duisburg-Essen, Essen, Germany; 5 Department of Gynecology and Obstetrics, Klinikum Duisburg and the University of Duisburg-Essen, Duisburg, Germany; Medical Faculty, Otto-von-Guericke University Magdeburg, Medical Faculty, Germany

## Abstract

Preeclampsia, a hypertensive disorder in pregnancy develops in 2–8% of pregnancies worldwide. Winter season and vitamin D deficiency have been associated with its onset.

**Objective:**

To investigate the influence of season on maternal vitamin D status and placental vitamin D metabolism.

**Methods:**

25-OH vitamin D and 1,25-(OH)_2_ vitamin D were measured in maternal serum obtained during the winter or summer months from 63 pregnant women at delivery (43 healthy, 20 preeclampsia). In a subgroup, mRNA expression of CYP24A1 (24-hydroxylase), CYP27B1 (1α-hydroxylase) and VDR (vitamin D receptor) were quantified by real time PCR in placental samples of 14 women with normal pregnancies and 13 with preeclampsia.

**Results:**

In patients with preeclampsia,25-OH vitamin D levels were lower, but differed significantly from controls only in summer (18.21±17.1 vs 49.2±29.2 ng/mL, *P*<0.001), whereas 1,25-(OH)_2_ vitamin D levels were significantly lower only in winter (291±217 vs 612.3±455 pmol/mL, *P*<0.05). A two-factorial analysis of variance produced a statistically significant model (*P*<0.0001) with an effect of season (*P*<0.01) and preeclampsia (*P* = 0.01) on maternal 25-OH vitamin D levels, as well as a significant interaction between the two variables (*P* = 0.02). Placental gene expression of CYP24A1, CYP27B1, and VDR did not differ between groups or seasons. A negative correlation between placental gene expression of CYP24A1 and CYP27B1 was observed only in healthy controls (r = −0.81, *P*<0.0001).

**Summary:**

Patients with preeclampsia displayed lower vitamin D serum levels in response to seasonal changes.The regulation of placental CYP24A1, but not of the VDR or CYP27B1 might be altered in preeclampsia.

## Introduction

Preeclampsia, a hypertensive disorder of pregnancy, affects about 2–8% of pregnancies worldwide [1]. It poses a threat to maternal and fetal health and often results in premature delivery of the child. The development of preeclampsia is linked to altered vitamin D metabolism [2], and might be a result from placental dysfunction [24]. However, the underlying cause of the development of preeclampsia remains elusive.

Worldwide, the onset of preeclampsia is more frequent during winter months, which in tropical countries corresponds to the rain season, than during any other season [3]. Since vitamin D deficiency is also more frequent during winter months, these findings point towards a potential role of vitamin D in the development of preeclampsia. Indeed, studies found lower 25-OH vitamin D serum levels as well as lower 1,25-(OH)_2_ vitamin D levels in patients with preeclampsia compared to healthy pregnant women of the same gestational age [2].

Vitamin D deficiency itself is common during pregnancy in the European and North American populations [4]–[6] and is associated with complications during the perinatal period [2] [7].

In the non-pregnant state, vitamin D status is regulated mainly by the enzymatic activity of the ‘1α-hydroxylase’ (CYP27B1), which converts 25-OH vitamin D to its active form 1,25-(OH)_2_ vitamin D (also called calcitriol), mostly at the renal site, and the ‘1,25-(OH)_2_ vitamin D 24-hydroxylase’ (24-hydroxylase, CYP24A1), which converts 1,25-(OH)_2_ vitamin D to the inactive 24,25-vitamin D.

During pregnancy, maternal 1,25-(OH)_2_ vitamin D levels are elevated [8]. It has been suggested that the physiological feedback regulation is uncoupled during pregnancy due to placenta-specific methylation of the promoter region of the 24-hydroxylase (CYP24A1), to allow for an increase in 1,25-(OH)_2_ vitamin D levels during the period of fetal development [9].

The placenta is thought to be the major site of vitamin D metabolism in pregnancy. The 1α-hydroxylase, the 24-hydroxylase, the 25-hydroxylase (CYP2R1), the vitamin D binding protein (VDB), and the vitamin D receptor (VDR) have all been detected either in trophoblast cultures or in freshly obtained, placental tissue [10]–[13]. Undoubtedly, the placenta is able to metabolize vitamin D, providing active 1,25-(OH)_2_ vitamin D *in vitro*. However, it is unclear to what extent placental vitamin D metabolism contributes to maternal vitamin D status in pregnancy.

Since placental dysfunction in preeclampsia might affect placental vitamin D metabolism, we aimed to investigate maternal and placental vitamin D metabolism in vitamin D deficient (winter) and sufficient (summer) season in women with and without preeclampsia.

## Results

### Maternal vitamin D and calcium status

Both delivery season and preeclampsia had a significant effect on maternal 25-OH vitamin D serum concentration (*P*<0.0001).

In patients with preeclampsia maternal 25-OH vitamin D serum levels were significantly lower compared to healthy controls during summer (18.21±17.1 vs 49.2±29.2 ng/mL, *P*<0.001).

**Figure 1:**
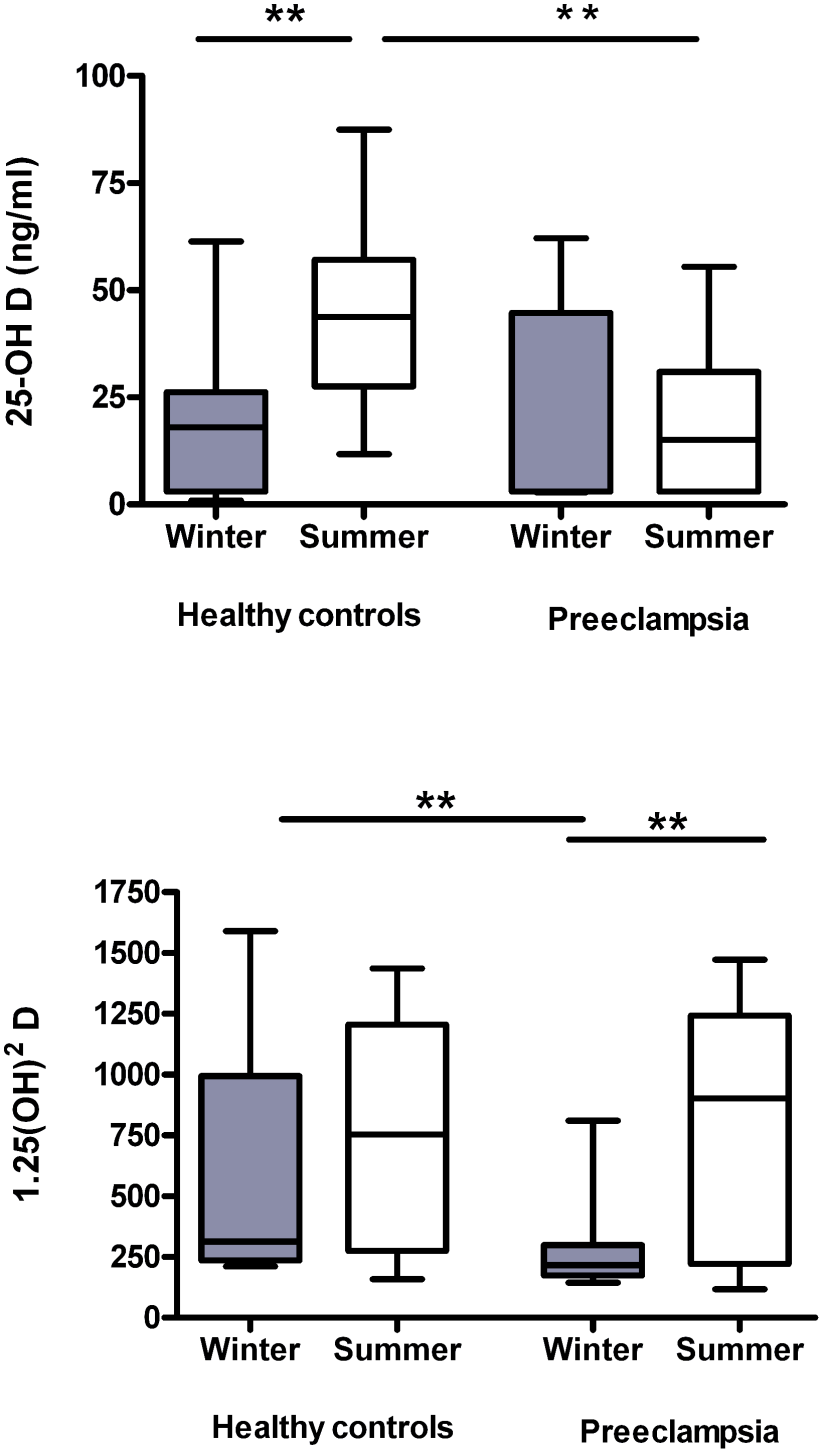
Boxplots showing the smallest observation (lower bar), lower and upper quartile (box), median (line in the box) and largest observation (upper bar) of a) maternal 25-OH vitamin D serum levels (ng/mL) in winter (grey box) and summer (open box). Maternal 25-OH vitamin D levels differ between patients with preeclampsia and healthy controls in the summer (** p<0.01). b) Maternal 1,25-(OH)_2_ vitamin D (pmol/mL) serum levels are similar in both groups, but significantly lower during winter months in patients with preeclampsia (** p<0.01).

In healthy controls, serum 25-OH vitamin D levels were higher in the summer than in the winter (49.2±29.2 vs 19.5±16 ng/mL). Surprisingly, these seasonal changes in 25-OH vitamin D levels were not present in patients with preeclampsia (18.21±17.1 vs 17.6±23.5 ng/mL), indicating a significant interaction between seasonality and pregnancy outcome on 25-OH vitamin D (*P* = 0.02, Regression Model). (Figure 1)

Maternal 1,25 -(OH)_2_ vitamin D levels were differently affected by season than 25-OH vitamin D levels: While no seasonal changes were detected in healthy controls (612.3±455.7 in winter vs.748.5±462 pmol/L in summer), patients with preeclampsia had significantly lower 1,25-(OH)_2_ vitamin D levels in the winter compared to the summer months (291±217 vs 815±478 pmol/L, *P*<0.01), and also compared to the levels of healthy controls (*P*<0.01) (Figure 1).

Maternal serum calcium levels did not differ between healthy controls (2.23±0.18 mmol/L) and patients with preeclampsia (2.13±0.17 mmol/L, Table 1). In both groups individual cases displayed serum calcium levels below the lower norm. Maternal serum calcium correlated positively with 25-OH vitamin D levels in healthy controls (r = 0.42, *P* = 0.015), but not in patients with preeclampsia. In the group of preeclampsia, maternal calcium levels correlated positively with the child-BMI at birth (r = 0.59, *P* = 0.023).

**Table 1:** Patient characteristics (mean + SD and (range)) for age, pre-pregnancy BMI, maternal calcium, 25- OH vitamin D, 1,25-(OH)_2_ vitamin D levels in serum, systolic (SBP) and diastolic (DBP) blood pressure on admission to the hospital, as well as child length, weight, and BMI standard deviation scores (SDS) at birth.

	All	Healthy controls	Preeclampsia
**Maternal AGE** (y)	31.9±5.6	32.2±5.4	31.3±6.1
	(20–43, 63)	(20–43, 43)	(21–41, 20)
**BMI**	28.5±6.6	28.5±7.1	28.5±5.3
	(18.5–44.1, 34)	(18.5–44.1, 24)	(22–36.8, 10)
**Calcium** (mmol/L)	2.19±0.18	2.23±0.18	2.13±0.17
	(1.7–2.9, 49)	(1.7–2.9, 32)	(1.9–2.4, 17)
**25-OHD** (ng/mL)	28.5±26.0	**33.3**±**27.3**	**18.2**±20.0*
	(0.98–132.8, 63)	**(0.1**–**132.8, 43)**	**(2.9**–**62.1, 20)**
**1,25-(OH)^2^ D** (pmol/mL)	643.5±456.4	671.7±457.5	582.6±461.2
	(118–1590, 57)	(158–1590, 39)	(118–1473, 18)
**SBP mmHG**	NA	**119.2 ± 6.**	172.3 ± 22.6**
		**0 (105**–**131, 21)**	**(135** ^#^–**220, 16)**
**DBP mmHG**	NA	**74.0 ± 8.4**	99.1 ± 10.1**
		**(60**–**88, 21)**	**(82** ^#^ **–118, 16)**
**Gestational week** (weeks)	37.6±2.7	**38.8**±**1.5**	**35.0**±2.9**
	(29–42, 63)	**(35.3**–**42, 43)**	**(29**–**40.3, 20)**
**Child Birth-Weight (g)**	2965±695	**3271**±**418**	**2269**±782**
	(4270–740, 63)	**(2050**–**4270, 43)**	**(740**–**3780, 20)**
**Child Birth-Weight SDS**	-0.50±1.	**−0.13**±**0.87**	**−0.15**±1.15**
	13 (−3.6–1.8, 60)	**(−2.5**–**1.8, 43)**	**(−3.6**–**0.8, 20)**
**Child Birth-Length (cm)**	49.7±3.6	**51**±**2**	**46**±5**
	(35–55, 60)	**(47**–**54, 43)**	**(35**–**55, 17)**
**Child Birth-Length SDS**	−0.51±1.13	**−0.16**±**0.73**	**−0.15**±1.5**
	(−4.7–1.43, 60)	**(**−**2.5**–**1.0, 43)**	**(−4.6**–**1.4, 17)**
**Child Birth-BMI SDS**	−0.30±1.12	**0.0**±**1**	−**1.16**±1 **
	(−3.0–2.25, 60)	**(−2.5**–**2.5, 43)**	**(−3**–**0.1, 17)**

In patients with preeclampsia, maternal 25-OH vitamin D levels are significantly lower than in healthy controls. * differs from healthy controls (*P<0.05, ** *P*<0.01) ^#^blood pressure obtained on admission to hospital was below the criterion for preeclampsia in one patient, despite preeclampsia with proteinuria and elevated blood pressure readings before and after admission.

### Placental gene expression of CYP24A1, CYP27B1, and VDR

The lowest mRNA levels between the three genes were found for CYP27B1. In few samples, CYP27B1 expression was below the detection limit. This affected 3of the 14 non-PE samples (21%), and 2 of the 13 PE samples (18%). Placental gene expression of neither CYP24A1 nor CYP27B1 nor VDR differed between seasons or between patients with preeclampsia and healthy controls (as determined by the Mann-Whitney test). Results are displayed in table 2.

**Table 2:** mRNA expression of placental CYP24A1, CYP27B1 and VDR (mean ± SD and (range) do not differ between patients with preeclampsia and healthy controls.

	All	Healthy controls	Preeclampsia
**Placental:**			
**CYP 24A1** (fold change)	1.2±0.7	1.2±0.7	1.1±0.7
	(0.1–2.8, 27)	(0.1–2.2, 14)	(0.3–2.8, 13)
**CYP 27B1** (fold change)	1.09±0.7	1.14±0.6	1.03±0.8
	(0.3–3.4, 22)	(0.4–2.1, 11)	(0.3–3.4, 11)
**VDR** (fold change)	1.2±0.6	1.1±0.4	1.3±0.8
	(0.5–2.7, 27)	(0.5–1.8, 14)	(0.5–2.7, 13)

**Figure 2:**
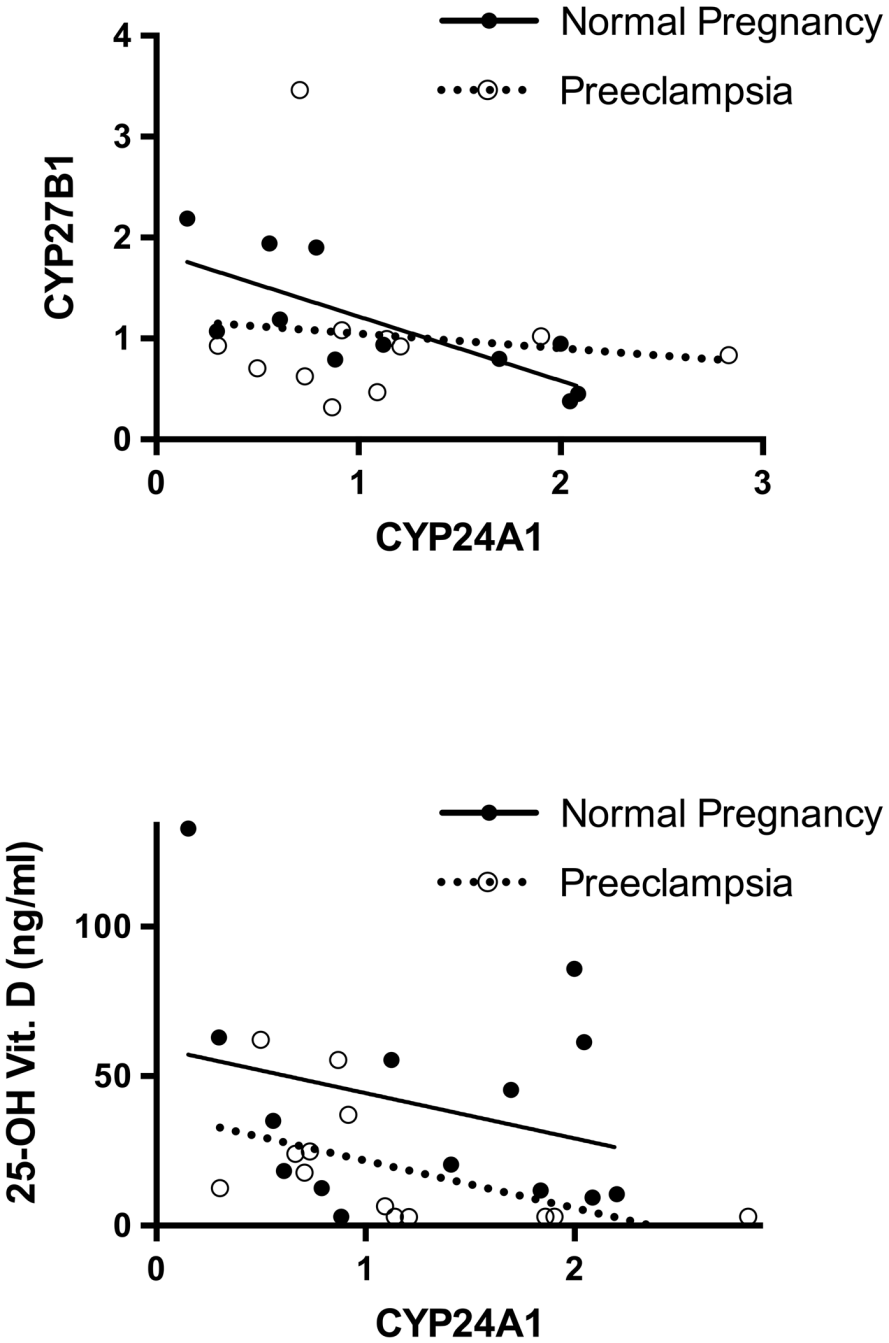
Correlation of placental mRNA expression of CYP24A1 (x-axis) and a) CYP27B1 or b) maternal 25-OH vitamin D (ng/ml) in healthy controls (black circles) and patients with preeclampsia (transparent circles). a) Placental gene expression of CYP24A1 correlates negatively with CYP27B1 expression in healthy controls (r = −0.81, *P*<0.0001, solid line) but not in the patients with preeclampsia (dotted line). b) CYP24A1 correlates negatively with maternal 25-OH vitamin D levels (r = −0.76, *P* = 0.01) in patients with preeclampsia (dotted line).

However, in healthy controls, placental gene expression of CYP24A1 negatively correlated with CYP27B1 expression (r = −0.81, *P*<0.0001) (Figure 2) and with maternal serum calcium levels (r = −0.8, *P*<0.01). This finding was not detectable in patients with preeclampsia (Figure 2).

Instead, in patients with preeclampsia, a negative correlation between placental gene expression of CYP24A1 with maternal 25-OH vitamin D levels (r = −0.76, *P* = 0.01) was observed (Figure 2).

In a stepwise regression analysis, adding placental CYP24A1, CYP27B1, and VDR gene expression to a model already containing season and pregnancy status, an additional effect of placental gene expression of CYP24A1 on maternal 25-OH vitamin D serum levels (partial r^2^ = 0,12) was detected. No such effects were seen on serum 1,25-(OH)_2_ vitamin D levels. Delivery-season and preeclampsia combined had no significant effect on maternal 1,25-(OH)_2_ vitamin D serum concentration.

## Discussion

Alterations in vitamin D metabolism in preeclampsia have been documented by many studies.

In this study, we report evidence supporting a role for vitamin D deficiency in the development of preeclampsia.

### Maternal vitamin D-status

Consistent with previous studies, we found significantly lower maternal 25-OH vitamin D serum levels in patients with preeclampsia than in healthy pregnant controls. Surprisingly, this was most apparent during the summer months, when 25-OH vitamin D levels increased in women with healthy pregnancies, but remained at deficient levels in patients with preeclampsia. It is feasible that low 25-OH vitamin D levels are a contributing factor to the development of preeclampsia. Conversely, it is also feasible that vitamin D deficiency is a result of preeclampsia.

Serum 1,25-(OH)_2_ vitamin D levels were higher (643.5±452.4 pmol/L) in this cohort of pregnant women compared to levels in non-pregnant females [8]. Other studies have demonstrated both lowered maternal 1,25-(OH)_2_ vitamin D and 25-OH vitamin D levels in preeclampsia compared to healthy controls [16] [17] [18]. In our study, maternal 1,25-(OH)_2_ vitamin D levels were significantly lower only during winter months in patients with preeclampsia. There was an impressive difference between summer and winter 1,25-(OH)_2_ vitamin D levels in this group. This finding may indicate that during winter months vitamin D deficiency becomes more severe in patients with preeclampsia resulting in the inability of the vitamin D metabolizing sites to provide sufficient levels of 1,25-(OH)_2_ vitamin D.

In the non-pregnant state, the kidney is the major site of calcitriol synthesis. During pregnancy, 1,25-(OH)_2_ vitamin D synthesis is uncoupled, resulting in increased amounts of the active hormone in maternal serum in healthy pregnancies [19]. Whether the high 1,25-(OH)_2_ vitamin D output is achieved entirely by the kidney or whether placental sites contribute to the production is not clear.

However, since both placenta and kidney are affected by preeclampsia, the pathophysiological changes in both these organs might result in an impairment of 1,25-(OH)_2_ vitamin D synthesis. This would be consistent with higher PTH levels in women affected by preeclampsia, indicating a chronic impairment of renal function [20]. It is a shortcoming of this study that no PTH levels of the participants were available. However, an impairment of kidney function resulting in elevated creatinine levels was not observed in this cohort.

Maternal 25-OH vitamin D levels did not correlate to any of the clinical data we assessed. However, maternal serum calcium levels positively correlated with child BMI at birth in pregnancies complicated by preeclampsia, indicating that calcium may have a beneficial role for fetal outcome in this condition.

### Placental gene expression of CYP24A1, CYP27B1, and VDR

There was no difference in placental gene expression of vitamin D metabolizing enzymes between patients with preeclampsia and healthy controls. Neither were differences detectable between samples that were taken during summer or winter respectively.

Particularly, no differences in CYP27B1 placental gene expression were found between healthy controls and patients with preeclampsia. It has previously been suggested that placental activity of the 1-αlpha hydroxylase, the product of CYP27B1, is responsible for the synthesis of 1,25(OH)_2_-vitamin D by the placenta. [9]. However, in about 20% of our samples, CYP27B1 mRNA expression was below the detections limits by real time PCR. In the remaining samples CYP27B1 mRNA was detectable, but at much lower levels than in e.g. in lymphocytes. This is a surprising finding and might point towards additional enzymatic pathways in the placenta, allowing for 1,25(OH)_2_-vitamin D synthesis.

Quantification of vitamin D metabolizing enzyme mRNA in freshly obtained placental tissue is rarely reported in published studies. Few studies show decreased CYP27B1 expression in cultured syncytiothrophoblasts of preeclampsia placentas compared to controls [11], and increased CYP27B1 protein levels in freshly obtained placentas from preeclamptic pregnancies compared to healthy pregnancies [10]. Increased mRNA expression of CYP27B1 was detected in placentas of preeclamptic women compared to at-term placentas of women with normal pregnancies. However, when compared to placentas of the appropriate gestational age, CYP27B1 expression was unaltered [21]. It is possible that CYP27B1 gene expression is differentially regulated across gestational age and that the time point chosen in our sampling protocol was not one during which aberrant expression levels between patients with preeclampsia and controls were present.

In healthy pregnancies, placental gene expression of CYP27B1 negatively correlates with CYP24A1 expression, indicating a functional regulation of the two opposing enzymes. In preeclampsia, this correlation is not detectable. Instead, there is a strong negative correlation between placental CYP24A1 gene expression and maternal 25-OH vitamin D serum levels in this condition. If the lowered maternal vitamin D status in preeclampsia is not solely attributable to reduced vitamin D intake and reduced renal activation of vitamin D, these findings may indicate that activation of the 25-hydroxylase, the product of CYP24A1, could play a role in the reduced serum levels of 25-OH vitamin D and 1,25-(OH)_2_ vitamin D in patients with preeclampsia. Halhali et al. (2014) showed a decrease in the production of 1,25-(OH)_2_ vitamin D in homogenates of preeclamptic placentas. As the authors discuss, this might be due to the fact that CYP24A1 is active and shifts vitamin D metabolism towards inactive forms (24,25 VDH) in preeclampsia [22].

Since placenta specific methylation of the promoter of CYP24A1 has been reported [9], alterations in methylation could be responsible for these changes. Further work investigating methylation status in placentas from pregnancies complicated by preeclampsia is needed.

Alternatively, inconsistencies in findings regarding the expression levels of vitamin D regulating enzymes in the placenta might be explained by their involvement in local immunological functions. Instead of contributing to maternal vitamin D levels, synthesis and metabolism of vitamin D in the placenta may serve placental requirements only. In the future, it would be valuable to assess 24,25-OH vitamin D levels in healthy versus preeclamptic pregnancies to investigate whether activation of CYP24A1 is related to the diminished 1,25-(OH)_2_ vitamin D levels observed in winter in patients with preeclampsia.

#### Limitations of the study

Preeclampsia poses a threat to maternal and fetal health. Delivery of the child is the only curative measure, and thus pregnancies affected by preeclampsia frequently result in premature delivery of the child. In our study, the mean gestational age at birth was 35.0 (29–40.3) gestational weeks in the group with preeclampsia, compared to 38.8 (35.3–42.4) gestational weeks in the group of healthy controls. It was not possible for us to investigate gestational-age matched placental samples from healthy pregnancies. Therefore – some of the differences reported in this study might be founded in the different gestational ages of the investigated groups.

## Summary

In summary, our study confirms that women with preeclampsia exhibit vitamin D deficiency compared to healthy controls. Interestingly, changes in maternal vitamin status are mediated by season, and seasonal changes differ in preeclampsia and healthy controls. Whether that is due to aberrant placental vitamin D metabolism is unknown. It is likely that the placental mRNA expression of CYP24A1 is dysregulated in preeclampsia and responsible for the observed seasonal differences in maternal vitamin D status.

Our study also indicates that higher levels of maternal serum calcium may have a beneficial effect on child BMI in preeclampsia. Since vitamin D deficiency during pregnancy is detrimental to maternal and fetal health, [6] [23] a sufficient vitamin D supplementation should be recommended for pregnant women.

## Patients and Methods

Samples and clinical data were obtained from a study cohort of 600 patients living in the Northern hemisphere. These patients were enrolled to identify prognostic markers for the development of preeclampsia at the Department of Gynecology and Obstetrics, UK-Essen, University of Duisburg-Essen, Germany during *January 2005 – December 2008*. The clinical data of the patients can be found in Gellhaus et al. [14]. Preeclampsia (PE) was diagnosed according to international criteria [15] [1]. Generally, PE was defined as a blood pressure of at least 140/90 mmHg on two occasions at least 6 h apart occurring after 20 weeks of gestation in women known to be normotensive beforehand and detectable urinary protein (proteinuria: ≥30 mg/dl or >1 by dipstick).

All included patients carried an intact singleton pregnancy. Patients were included after written informed consent was obtained. The study was approved by the local ethics committee [Ehik-Kommission der Medizinischen Fakultät Essen, votum No. 03-2157 (April 2003) and 08-3684 (July 2008)].

From this cohort, serum samples of all women who delivered during the summer months (June, July, and August) or during the winter months (December, January, and February) were included into the analyses (N = 63). Of the 63 women, 20 had developed preeclampsia during pregnancy (PE) and 43 had normal pregnancies (non-PE).

Clinical data, including: biochemical data (maternal serum calcium, phosphate, alkaline phosphatase, creatinine), parity, ethnicity, maternal pre-pregnancy weight, maternal height, maternal blood pressure, presence of proteinuria, birth modus, child birth weight, child birth height, and Apgar scores, were retrieved from patient charts.

### Patient characteristics

Patients with preeclampsia did not differ from healthy controls with respect to age, pre-pregnancy BMI, parity, or serum levels of calcium, alkaline phosphatase, or creatinine.

As expected, birth occurred at an earlier gestational age in patients with preeclampsia and, accordingly, child birth weight, child birth length, and child birth BMI were significantly lower in offspring of these mothers (*P*<0.001, Table 1).

33% of the infants born to mothers with PE were small for gestational age (SGA) as defined by a birth weight below the 3^rd^ percentile for the appropriate gestational age (vs 4% in the non-PE group) and 60% displayed a birth weight below the 10^th^ percentile for the appropriate gestational week, versus 9% in the non-PE group. Apgar scores at 5 minutes were lower in infants of PE mothers (8.6±1.5) vs non-PE mothers (9.6±0.7, *P*<0.001)

Blood pressure was significantly higher in women with preeclampsia compared to healthy controls (*P*<0.001, Table 1). All patients with preeclampsia displayed proteinuria (99±68.6, range 35–255 mg/dl). Of the 43 healthy controls, 39 were Caucasian and 4 of African descent. Of the 20 women with preeclampsia, 18 were Caucasian and 2 of African descent. See Table 1 for patient characteristics.

### Immunoassays

Maternal 25 OH-vitamin D serum levels were assessed using a commercial enzyme-linked immunosorbent assay (25-OH-vitamin D ELISA, Immundiagnostik, Bensheim, Germany). Intra-assay variation was 9.6% at 20.1 nmol/l, inter-assay variation was below 13% for samples measuring 33–110 nmol/l.

Maternal 1,25-(OH)_2_ vitamin D serum levels were assessed using a commercial enzyme immunoassay [1,25-(OH)_2_ vitamin D EIA, Immunodiagnostic Systems Limited (IDS Ltd.), Boldon, UK]. Intra-assay variation was below 11% and inter-assay variation was below 17% for samples measuring 19–152 pmol/l.

### Real time PCR

Total placental RNA was isolated from the placental tissues of 14 normal pregnancies and 13 pregnancies complicated by preeclampsia. Only chorionic tissue from the central part of the placenta, close to the center of the insertion of the umbilical cord, was collected and contamination with maternal decidua and amniotic membranes was excluded by morphological observation. Tissues were frozen in liquid nitrogen and stored at −80°C until extraction of total RNA was performed using the E.Z.N.A.Total RNA kit (Omega Bio-Tek, Norcross, USA). For the removal of DNA contaminants, 2 µg RNA was incubated with 1 U/µl DNase I (Invitrogen, Karlsruhe, Germany) and 2 µg RNA was used as a template for cDNA synthesis using the SuperScript II pre-amplification system, according to manufacturer instructions (Invitrogen, Life Technologies, CA, USA).

Placental expression of CYP24A, CYP27B, and VDR was assessed using real time PCR and quantified against the β-actin signal. The following primer sequences (sense-antisense) were used for Cyp27B1: ggaaggcgaagaatggcaaagg – tcgcagactacgttgttcagggttc; CYP24A1: tctggaaagggggtctcaagaaaca – accgactcaaaggaacccaacttca; VDR: ggacctgtggcaaccaagactacaa – ttcagtcccacctggaacttgatga; and Actin: catcctcaccctgaagtaccccatc – agccacacgcagctcattgtagaag. Fold change was calculated by normalizing to the mean gene expression of healthy controls.

### Statistical analysis

The effect of season and preeclampsia on serum vitamin D metabolite concentrations was tested by a two-factorial ANOVA. The influence of placental expression of CYP24A1, CYP27B1, and VDR was assessed by a multiple stepwise regression analysis, adding these variables to a model containing season and pregnancy outcome. The Shapiro Wilk test was used to test for normal distribution of the data. The hypothesis for normal distribution was rejected at α<0.1 (Proc univariate, SAS 9.4 SAS Institute Inc., Carey, NC, USA, 2008). Associations between single variables were described by the Spearman correlation. Differences between group means and reference cohorts were assessed by the Mann-Whitney test. Statistical significance was assumed at *P*<0.05. Data are given as mean ±1 SD. Data analysis was performed using SAS statistical software (SAS System, release 9.2, SAS Institute Inc., Carey, NC, USA, 2008).
